# Mechanistic Insights into Nano-Maillard Reaction Products Regulating the Quality of Dried Abalones

**DOI:** 10.3390/foods14152726

**Published:** 2025-08-04

**Authors:** Jialei Shi, Hongbo Ling, Yueling Wu, Deyang Li, Siqi Wang

**Affiliations:** 1 School of Biological Engineering, Dalian Polytechnic University, Qinggongyuan 1, Dalian 116034, China; shijialei1998@163.com (J.S.); 18153836850@163.com (H.L.); 13470125919@163.com (Y.W.); 2 State Key Laboratory of Marine Food Processing and Safety Control, National Engineering Research Center of Seafood, Collaborative Innovation Center of Seafood Deep Processing, Dalian Polytechnic University, Dalian 116034, China; 3 Liaoning Province Key Laboratory for Marine Food Science and Technology, Dalian Polytechnic University, Dalian 116034, China; 4 School of Food Science and Technology, Dalian Polytechnic University, Dalian 116034, China

**Keywords:** Maillard reaction products, Abalone, drying process, moisture mobility, texture profile analysis

## Abstract

Broth cooking is a traditional pretreatment and ripening strategy for high-commercial-value dehydrated marine food, effectively enhancing its texture and rehydration properties. In this work, we characterized the structural information of Maillard reaction products (MRPs) derived from beef scrap stock and investigated their effects on the texture and rehydration performance of dehydrated abalone. The optical and structural properties of the MRPs were analyzed using X-ray photoelectron spectroscopy (XPS), X-ray diffraction (XRD), transmission electron microscopy (TEM), and fluorescence spectroscopy. These MRPs showed osmosis in abalone processing including pretreatment and drying. Low-field nuclear magnetic resonance (LF-NMR) results revealed that MRP pretreatment improved the moisture migration and physicochemical properties of dehydrated abalone. These findings suggest that MRPs, owing to their high osmotic efficiency and nanoscale size, could serve as promising food additives and potential alternatives to traditional penetrating agents in the food industry, enhancing the rehydration performance of dried seafood and reducing quality deterioration.

## 1. Introduction

Abalone (*Haliotis discus hannai Ino*) is a highly valued marine resource prized for its flavor and nutritional richness. However, its preservation under atmospheric conditions remains challenging. Drying is an effective method for extending abalone’s shelf life and facilitating its transportation at ambient temperature [1]. Beyond preservation, dehydration also enhances abalone’s flavor and aroma. The quality of dried abalone is largely determined by its shrinkage degree and moisture distribution. Hot air drying, the most common dehydration method for abalones, is cost-effective and convenient but often compromises the functional properties of the final product [2]. To minimize these adverse effects, appropriate pretreatment methods are essential. One promising approach is osmotic-assisted drying, which utilizes high-pressure osmotic solutions to improve drying rates and moisture uniformity. This method facilitates moisture infusion during rehydration, converting free water into intermediate moisture and thereby enhancing the mouthfeel of rehydrated products [3].

Maillard reaction products (MRPs) are nanometer-sized spherical granules with antioxidant and antibacterial properties, naturally produced in food processing [4]. The synthesis of MRPs is environmentally friendly, utilizing natural and renewable low-cost materials. For instance, fluorescent MRPs have been successfully extracted from various food sources, including beer [5], Oolong tea [6], and bread [7]. Protein and polysaccharides serve as key reactants in the Maillard reaction, and beef scraps such as bovine connective tissue and bone are commendable materials for MRP production. However, there is a risk of acrylamide emissions during the violent synthesis process [8], making hydrothermal synthesis a more controlled and practical alternative due to its ease of implementation and ability to produce MRPs with high quantum yield [9]. Controlled MRPs in food processing contain no heavy metal ions and are considered relatively safe. Their low toxicity, strong fluorescence, excellent water solubility, and stable chemical properties contribute to their high biocompatibility, making nanoscale MRPs particularly advantageous for protein-based food applications [10]. High-penetration MRPs extracted from roast beef have been reported to be low-toxicity nanoparticles [11]. Despite their potential, the use of non-toxic MRPs as additives to enhance the quality of dried and rehydrated agricultural products remains underexplored.

In this study, we propose that MRPs derived from beef scrap stock could enhance the quality of abalones when applied as penetrating agents to the drying process. The physicochemical characteristics of MRPs, including their morphology, chemical composition, and fluorescence properties, are systematically characterized. Furthermore, the effect of MRP treatment on the relaxation behavior, textural properties, and rehydration capability of dried or rehydrated abalone is investigated. Based on these comprehensive analyses, we establish specific correlations between processing parameters and texture profile analysis (TPA) results.

## 2. Materials and Methods

### 2.1. Synthesis of MRPs

Beef scraps were washed with distilled water several times, chopped into small pieces, mixed with distilled water in a 1:1 (*w*/*w*) ratio, and subjected to 120 °C thermal processing in a pressure cooker for 3 h. Following thermal treatment, the suspension containing MRPs was centrifuged at 8000 rpm for 20 min to remove the insoluble particles. The supernatant was purified with an activated D101 macroporous adsorption resin column (Yuanye Biotechnology Co., Ltd., Shanghai, China) and eluted with distilled water. The elution was collected and concentrated by a lyophilizer (ZLGZ-10, Telstar, Barcelona Spain), yielding MRP powder for further use.

### 2.2. Characterization of MRPs

Transmission electron microscopy (TEM) analysis of the MRP powders was carried out on a JEM2100 UHR (Japan Electronics Co., Ltd., Tokyo, Japan) to determine the morphology and size of the MRPs with an electron energy of 200 kV and a magnification of 50,000×. The chemical composition of the MRP powders was identified using the ESCALAB 250 X-ray Photoelectron Spectroscopy (XPS) system (Thermo Scientific, Waltham, MA, USA) with Al Ka excitation (1486.7 eV) as the source of the monochromator. The ultraviolet–visible (UV-vis) absorption spectra of the MRPs (1 mg/mL) were recorded using a Lambda 35 (Perkin Elmer, Waltham, MA, USA); appropriate blank corrections were performed using distilled water.

### 2.3. Calculation of Quantum Yield and Lifetime

The quantum yield of MRPs was measured using a FluoroMax-4 spectrofluorometer (Horiba Scientific Co., Albany, NY, USA) at 376 nm with an excitation laser. A 0.1 mol/L quinine sulfate solution was used as a reference standard with quantum yield (Փr) = 0.54 at 376 nm. To consider the MRPs’ effects on the absorbance values of each solution in the cuvette, the values were controlled under 0.1. The quantum yield (Փ) of MRPs was calculated according to Equation (1):

(1) $$\Phi =\Phi_{r}IA_{r}\eta^{2}/IrA\eta_{r}^{2}$$

Herein, Փ is the quantum yield of the MRPs, I is the measured corrected emission intensity, A is the absorbance at the excitation wavelength, and ƞ is the refractive index of the solution. The subscript r represents the reference fluorophore of a known standard solution. The fluorescence lifetime (τ) of the MRPs was calculated based on Equation (2):

(2) $$R(\tau )=B_{1}e(\tau /\tau_{1})+B_{2}e(\tau /\tau_{2})$$

Herein Bn is the fractional contribution of the time-resolved decay lifetime of τi.

### 2.4. Cytotoxicity Assessment of MRPs

Normal rat kidney (NRK) cells were selected to assess the MRPs’ cytotoxicity using the method described by Yin et al. [7]. Simply put, NRK cells were seeded at a density of 1 × 10^5^ cells/mL in 96-well plates and cultured for 24 h. Following 12 h of treatment with MRP solutions, cytotoxicity was assessed using the Methylthiazolyldiphenyl-tetrazolium bromide (MTT) assay. A volume of 20 μL of 5 g/L MTT solution was added to each well and incubated for 4 h. Subsequently, the supernatant was carefully removed, and 150 μL of dimethyl sulfoxide was added to each well to dissolve the formazan crystals. The plates were gently shaken, and the absorbance was measured at 490 nm using a microplate reader (F50, TECAN, Männedorf, Switzerland). Cell viability was calculated as a percentage relative to untreated control cells.

### 2.5. Abalone Sample Preparation

Abalones in the shell were purchased from the local seafood market in Dalian, China. Specimens with an average body weight of 30 ± 3 g were selected for experiments. After their shells and viscera were removed, the blank group of abalone was kept in deionized water, and the experiment group was soaked in 0.1 g/L MRP aqueous solution. Both groups underwent a two-stage thermal treatment process of 60 °C for 90 min and 80 °C for another 30 min in a precisely controlled water bath. The abalones treated with water and MRPs were designated as Con and MRPs, respectively.

### 2.6. Determination of Drying Kinetics and Rehydration Ratio

The boiled abalones were dried in an electric air-drying oven (Shanghai Yiheng Technical Co., Ltd., Shanghai, China) at 60 °C for 24 h. During the drying process, samples were weighed every 2 h. Drying was terminated when three consecutive weight measurements exhibited less than 2% variation, indicating moisture equilibrium. The moisture ratio of the abalone was calculated as the initial moisture content of water-bathed abalone based on the weight of dried abalone. The drying rate was calculated as the moisture ratio based on the time interval. The dried abalones treated with different boiling liquids and solvents were named Dried and Dried-MRPs.

For rehydration, the dried abalone samples were rehydrated in deionized water or MRPs at 4 °C for 100 h, with their weights recorded every 2 h. Rehydration was considered complete when the difference among three consecutive measurements was less than 2%, signifying equilibrium. The rehydration ratio of the abalone was calculated as the initial weight of rehydrated abalone based on the weight of dried abalone. Samples treated with water throughout both the boiling and rehydration stages were named “Rehydrated”. Samples treated with water during the boiling stage and MRPs during the rehydration stage were named “Rehydrated-MRPs”. Samples treated with MRPs throughout both the boiling and rehydration stages were named “Rehydrated with MRPs”.

### 2.7. NMR Transverse Relaxation Measurements

Transverse relaxation was performed using an NMR analyzer (Suzhou Niumag Analytical Instrument Co., Suzhou, China) equipped with a 0.5 T permanent magnet at 32 °C, according to previous studies [12]. The decay signal of abalone was collected using the Carr–Purcell–Meiboom–Gill (CPMG) sequence at a resonance frequency of 23.1 MHz. The pulse sequences with 90^o^ and 180^o^ pulses were 13.0 and 26.0 µs, scanned with 2000 echoes and 8 scan repetitions. The decay data were fitted by MultiExp Inv Analysis software (Suzhou Niumag Analytical Instrument Co.) to acquire the T_2_ spectra.

### 2.8. Texture Profile Analysis

Texture profile analysis (TPA) of the abalone was carried out by a TA.XT plus texture analyzer (Stable Micro Systems, London, UK). Abalones were cut into 10 × 10 × 10 mm samples, ensuring parallel surfaces for consistent testing. A 50 mm diameter cylindrical aluminum probe (P/50) was employed to compress samples to 70% deformation. The curves were executed with a pre-speed, test speed, and post-speed of 1.0, 1.0, and 2.0 mm/s. The relaxation time between two contractions was 5 s. Hardiness, springiness, cohesiveness, chewiness, and resilience were computed using texture expert software version 5.1.1.0 (Stable Micro Systems Ltd, London, UK).

### 2.9. Thiobarbituric Acid-Reactive Substances

Thiobarbituric acid-reactive substances were determined followed the method of Cao et al. [13] to represent the degree of lipid oxidation in the abalones. Samples of 0.5 g rehydrated abalone were homogenized and reacted with a 10 mL reaction mixture containing 0.375% thiobarbituric acid (*w*/*v*), 15% trichloroacetic acid (*w*/*v*), and 0.25 mol/L HCl. The mixture was heated in a boiling water bath for 20 min, and the supernatant’s absorbance was measured at 532 nm using a spectrophotometer.

### 2.10. Statistical Analysis

Data were analyzed using SPSS software (IBM SPSS statisrics 19, IBM Corporation, Chicago, CA, USA ). with one-way analysis of variance at *p* < 0.05. The experiments were conducted in triplicate and the results are presented in the form of the mean ± standard deviation. Image drawing was accomplished using Origin software (Pro.2024, Origin Lab Co., Northampton, MA, USA). Heatmap results were analyzed and visualized through dimensionality reduction using R programming. Neural network data processing and modeling were performed using SPSS software.

## 3. Results and Discussion

### 3.1. Structural Properties and Cytotoxicity of the MRPs

**Figure 1:**
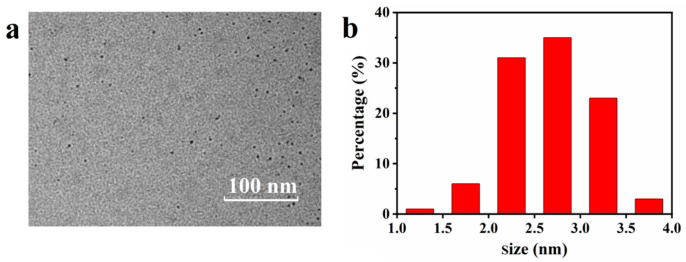
(**a**) High-resolution TEM image and (**b**) particle size distribution of the MRPs extracted from beef scrap stock.

The MRPs extracted from beef scrap stock under high-temperature and high-pressure processing were characterized using high-resolution TEM. As shown in Figure 1a, the MRPs exhibited a uniform spherical morphology with nanoscale dimensions. A quantitative analysis of 100 particles revealed a size distribution ranging from 1.0 to 5.0 nm, with an average particle diameter of 2.68 ± 0.47 nm (Figure 1b). The nanoscale dimensions of these MRPs enhance their permeability, making them particularly effective for subsequent applications. During the broth processing, the beef scraps underwent simultaneous hydrolysis and carbonization, involving initial pyrolysis oxidation of larger particles during the pre-synthesis stage followed by progressive disintegration and carbonization into nanometer-sized MRPs [14]. Prolonged broth processing facilitated extensive protein hydrolysis, thereby increasing the availability of free amino acids for subsequent Maillard reactions. This thermal treatment significantly enhanced both the yield and the physicochemical properties of the resulting nanometer-scale MRPs [15]. These occurring MRPs represent complex polymeric systems composed of proteins, polyphenols, and oligosaccharides, which commonly form during the thermal processing of various food products including coffee, cocoa, bakery items, and meat stocks [16,17]. The unique functional properties and demonstrated biological activities of MRPs suggest promising applications in food preservation and sensory quality enhancement.

**Figure 2:**
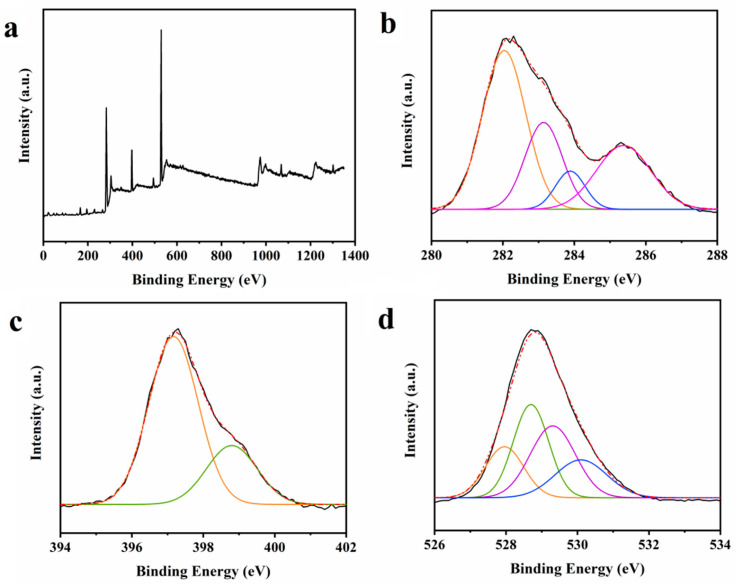
The surface elemental composition and chemical bonding states of the MRPs. (**a**) XPS survey spectrum, (**b**) C1s spectra, (**c**) N1s spectra, and (**d**) O1s spectra.

XPS was employed to investigate the surface elemental composition and chemical bonding states of MRPs extracted from beef scrap stock (Figure 2). The survey spectrum revealed characteristic C1s, N1s, and O1s peaks at binding energies of 283 eV, 397 eV, and 529 eV, respectively, with corresponding atomic percentages of 38.75%, 14.55%, and 46.69% (Figure 2a). The XPS results indicated the MRPs were rich in carbon, nitrogen, and oxygen derived from the proteins and polysaccharides in the beef scraps. In detail, Figure 2b shows the high-resolution binding energy at 282.0, 283.1, 283.9, and 285.4 for sp2-hybridized carbon and sp3-banded carbon, respectively. The N1s spectrum (Figure 2c) fitted with two Gaussian components with maxima located at 397.2 and 398.8 eV, indicating the presence of pyrrolic N and pyridinic N, respectively, further indicating the transformation of proteinaceous nitrogen into heterocyclic nitrogen forms during broth cooking [18,19]. This transition suggests thermal stabilization of nitrogen-containing moieties through the formation of aromatic structures [20]. Furthermore, three peaks at 528, 531.4, and 532.6 eV correspond to O-H, C=O, and C-O in the high-resolution O1s spectra (Figure 2d) of the MRPs [21]. Research conducted by Han et al. revealed that the Maillard reaction significantly alters the structure of proteins, which is directly reflected in the changes in groups and directly affects the functional characteristics of MRPs [22].

### 3.2. Optical Properties of the MRPs

**Figure 3:**
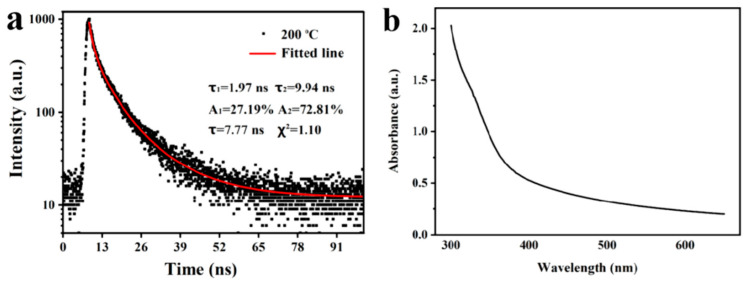
(**a**) Time-resolved fluorescence attenuation and fitted curve; (**b**) UV-vis absorption spectrum of the MRPs.

The optical properties and the non-mono-exponential decay of the time-resolved fluorescence attenuation of MRPs extracted from beef scraps are shown in Figure 3. The photoluminescence decay curve of the MRPs was well fitted by a bi-exponential function. The average fluorescence lifetime of the MRPs was 7.77 ns, and the fluorescence quantum yield of the MRPs was determined to be 72.81% using quinine sulfate as a reference standard. The absorption spectrum of the MRPs revealed prominent peaks within 300~700 nm, with minimal variations in the visible region. A weak absorption peak at 326 nm was observed, which is associated with n-π* transitions of C=O and C=N groups in pyrazines, pyridines, pyrroles, and aldehydes [23].

**Figure 4:**
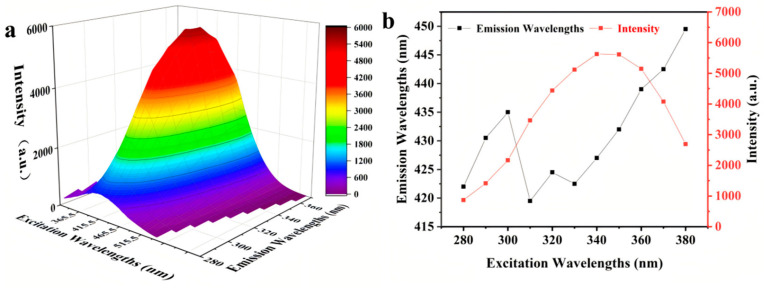
(**a**) The 3D fluorescence spectrum and (**b**) the emission wavelength and intensity of the MRPs under 280~370 nm excitation wavelengths.

The fluorescence emission spectrum exhibited a distinct red shift, with maximum emission wavelengths increasing from 423 nm to 450 nm as excitation wavelengths varied from 330 nm to 380 nm (Figure 4). The fluorescence intensity demonstrated wavelength-dependent behavior, showing continuous enhancement between 280 nm and 350 nm excitation, followed by a gradual decrease at higher excitation wavelengths (350–380 nm). These optical properties originate from the specific molecular structures of the browning products formed during thermal broth processing. UV-vis absorption analysis revealed a characteristic peak at 294 nm, indicating the presence of intermediate Maillard reaction products, including Strecker degradation-derived aldehydes and ketones. However, extended pressure cooking (360 min at 121 °C) promoted further reaction pathways, resulting in minimal residual aldehydes/ketones and substantial formation of heterocyclic compounds including pyridines, furans, pyrans, and furfurals, as evidenced by strong characteristic signals in the MRPs’ spectra [24]. These findings are consistent with the fluorescence patterns observed in MRPs derived from traditional Chinese medicine preparations [25].

### 3.3. Drying Kinetics and Rehydration

**Figure 5:**
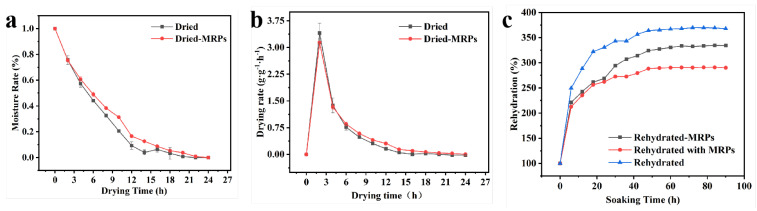
The (**a**) moisture ratio of abalone during drying, (**b**) drying rate of abalone, and (**c**) rehydration rate of dried abalone. Dried abalones treated with different boiling liquids and solvents were named Dried and Dried-MRPs. Samples treated with water throughout both the boiling and rehydration stages were named “Rehydrated”. Samples treated with water during the boiling stage and MRPs during the rehydration stage were named “Rehydrated-MRPs”. Samples treated with MRPs throughout both the boiling and rehydration stages were named “Rehydrated with MRPs”.

Toxicological assessment revealed a minimal impact of the MRPs on cellular proliferation (Figure S1), supporting their safety for the experimental applications described herein. Water evaporates from the inner region to the outer surface of a sample under thermal energy and airflow; however, the dried tissue structure of the sample surface may hinder water transport during drying [26]. Figure 5a,b depict the moisture content and drying rate of abalone samples, comparing MRP-treated samples to those that were conventionally pretreated. Both treatment groups exhibited characteristic drying behavior of an initial rapid dehydration phase followed by a gradual decline until reaching a constant weight. During the initial drying phase, boiled abalone tissue contained substantial amounts of water that was readily removed through thermal conduction and convection. The temperature of the sample rapidly increased to the actual temperature and remained constant during the whole drying stage, so the sample dehydrated quickly in the initial stage [27]. MRPs improved the resistance of the internal boundary layer of the sample, helped the abalone tissue to lock in water, increased the water holding capacity of the abalone tissue, and thus affected the drying power curve of abalone.

The time required to reach the equilibrium moisture content differed significantly between treatments. Control samples (pure water-boiled) achieved moisture equilibrium after 20 h, whereas MRP-treated samples required 24 h to reach stabilization. Abalone samples treated with pure water showed a turn in the drying rate and a slower rate of water content decline at 14 h, whereas the MRP-treated abalone showed a turn at 12 h. The dehydration rate of the samples was increased under the same conditions by MRPs, so the drying and dehydration times of the samples were reduced. As the water content continued to decrease, the rate of dehydration also decreased, and the drying rate slowed down.

Rehydration capacity serves as a critical quality parameter for evaluating drying methods, given that most dried seafood products undergo rehydration prior to consumption [28]. This complex physical process is influenced by multiple factors including pretreatment conditions and drying parameters [29]. When the drying process maintains optimal microstructural integrity, the rehydrated product cannot theoretically achieve moisture content levels comparable to those of its original state [30]. The degree of rehydration ratio serves as a sensitive indicator of structural preservation during dehydration, with higher rehydration capacity correlating with minimized cellular collapse and maintained porosity in the dried matrix. Figure 5c clearly shows that the rehydration ratio was obviously affected by MRPs. The rehydration kinetics revealed distinct differences between treatment groups. Rehydrated and Rehydrated-MRPs demonstrated a significantly higher rehydration rate (*p* < 0.05), achieving maximum ratios of 334% and 367% at 90 h rehydration, respectively. This enhanced rehydration ratio in Rehydrated suggests better preservation of microstructural integrity in these samples, as intact tissue architecture facilitates more efficient water absorption through capillary action and osmotic processes. The superior rehydration capacity observed in Rehydrated-MRPs samples could potentially result from the enhanced permeability characteristics of MRPs, which originate from their nanoscale dimensions and surface functional groups. In contrast, Rehydrated with MRPs samples demonstrated a comparable but reduced rehydration ratio. This performance limitation likely results from structural damage. Although MRPs effectively permeated the abalone tissue matrix during primary thermal processing, their subsequent presence in the rehydration medium paradoxically inhibited water absorption. Notably, MRP treatment inhibited the lipid oxidation of rehydrated abalone, which was particularly evident in the abalone with osmotic pretreatment before drying (Figure S2). The previous evidence indicates that MRPs can effectively slow down lipid oxidation, which may be related to the antioxidant capacity exhibited by MRPs [13,31].

### 3.4. NMR Analysis of Boiled, Dried, and Rehydrated Abalone

**Figure 6:**
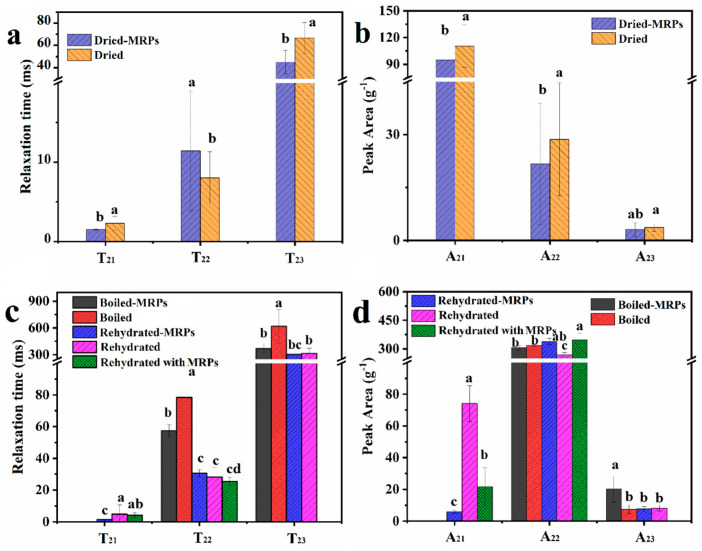
Relaxation times and peak areas of dried and rehydrated abalone. (**a**) Relaxation times of dried abalone, (**b**) peak areas of dried abalone, (**c**) relaxation times of rehydrated abalone, and (**d**) peak areas of rehydrated abalonelone. Different lowercase letters indicate significant differences between groups (*p* < 0.05).

Subtle changes in moisture in foods have been ignored for a long period. Low-field nuclear magnetic resonance (LF-NMR) is a fast and non-destructive method for moisture monitoring and has been used as a sensitive technology to describe moisture mobility in agriculture products during food processes [32]. The CPMG sequence was implemented to observe the location and populations of moisture, revealing the difference in muscle tissues based on proton changes. For instance, LF-NMR has excellent potential for monitoring subtle water dynamic changes in aquatic product processing, such as that of Spanish mackerel [33] and tilapia [34]. The effect of MRPs on the moisture distribution obtained from the CPMG sequence in abalone is shown in Figure 6, and the specific T_2_ spectra are shown in Figure S3. Moisture components with different environments were partitioned into bound water, immobilized water, and free water and labeled as T_21_, T_22_, and T_23_ according to previous studies of abalone and sea cucumber. The first peak at 1–10 ms of the relaxation time was associated with bound water closely attached to the macromolecules, such as moisture bound with protein molecules. T_22_ was assigned to immobilized water associated with or trapped within highly organized structures at 10–100 ms. T_23_, with the longest relaxation time, was considered to be the moisture in the extra-myofibrillar lattice [35]. The peak area of the T_2_ curve in abalone was assigned to A_21_, A_22_, and A_23_, representing the populations of T_21_, T_22_, and T_23_, respectively.

The moisture between fibers was reserved more and combined tightly in boiled abalone treated with MRPs. There was no T_21_ in the boiled abalone samples, where the immobilized water may have been covered by a high percentage of bound water. Little changes were observed in the total population of T_2_ (A_22_+A_23_) in boiled abalone; most noteworthy, moisture was tightly connected with boiled abalone treated with MRPs, as compared to untreated boiled abalone. Before drying, the T_2_ relaxation curves showed main peaks with relaxation times of 1.49 and 2.30 ms in boiled abalone and abalone boiled with MRPs; the signal of the main peak (T_21_) increased to 94.98 and 110.35 throughout the drying process of abalone pretreated and untreated with MRPs. A_21_ and A_22_ showed a significant and dramatic decrease for the dried abalone dehydrated after pretreatment with MRPs of high permeability (Figure 6b,d). After the drying process, the T_22_ component was reduced, and the population of bound water was recognized as the predominant component in dried abalone. While under the action of the drying process, the fiber in the dried abalone was much more connected with moisture, especially for the abalone treated by boiling in MRPs. In abalone treated with MRP broth, the reduction in the tightly bound moisture content, coupled with the inherent permeability of the MRPs, facilitated internal moisture transfer, leading to an earlier occurrence of the transition point phenomenon.

In the previous work, the results showed that a lower concentration of osmotic solution assisted in improving the rehydration rate to a certain extent. However, increasing the concentration of the osmotic solution had little effect on the rehydration ratio [36,37]. The abalone treated with MRPs had a marked T_22_ content, not only in the rehydrated abalone that had been boiled with MRP solution but also in normal abalone rehydrated with MRP solution. The T_22_ values increased from 8.01 to 28.27 ms with the rehydration process in abalone rehydrated with water, indicating that the contracted fiber in the abalone expanded with increasing T_22_. For untreated abalone, rehydration with MRP solution resulted in lower mobility of inter-fiber water, and abalone boiled with MRPs exhibited a reduction in its immobilized water population. However, MRPs with strong permeability led to a decrease in moisture mobility with tight compaction in the rehydrated abalone. MRPs were infused into abalone tissue with the assistance of heat and mass to overcome the difficulty of moisture entering the shrunken tissue during the rehydration process, increasing the moisture content and decreasing water mobility in the rehydrated abalone.

### 3.5. Evolution of TPA Parameters Related to Boiling and Rehydrating Process in Dried Abalone

The effect of MRPs on abalone texture was evaluated using TPA, focusing on hardness, springiness, and chewiness. Osmotic-assisted drying has been reported to induce a more compact structure and minimize muscle decomposition in dried fish, which could influence textural properties [38]. MRP pretreatment revealed a minimal impact on elasticity, as osmotic penetration had negligible structural effects on the boiled samples [39]; such effects depend on the properties of myofibrillar, sarcoplasmic, and connective tissues [40,41]. In MRP-treated abalone, the increased hardness and chewiness were attributed to water loss and tissue structure shrinkage induced by osmotic effects during the boiling process. This effect persisted throughout the drying process; consequently, the hardness values of conventionally and MRP-boiled abalone after drying were measured at 12,648.37 and 14460.47, respectively. The MRP-treated abalone demonstrated significantly enhanced rehydration capacity and moisture absorption, likely due to synergistic interactions between moisture and protein components. Throughout the rehydration process, the hardness of abalone boiled with MRPs was 10,640.57, which further increased to 13,209.61 when rehydrated in MRP solution. In contrast, chewiness parameters showed no significant variation between MRP-treated and control samples following rehydration. The incorporation of MRPs during boiling induced subtle but potentially important textural modifications. This pretreatment may enhance both moisture retention and texture in subsequent processing stages, as evidenced by the observed improvements in rehydration capacity and hardness parameters.

### 3.6. Main Stage and Factor Analysis of Abalone Processing

**Figure 7:**
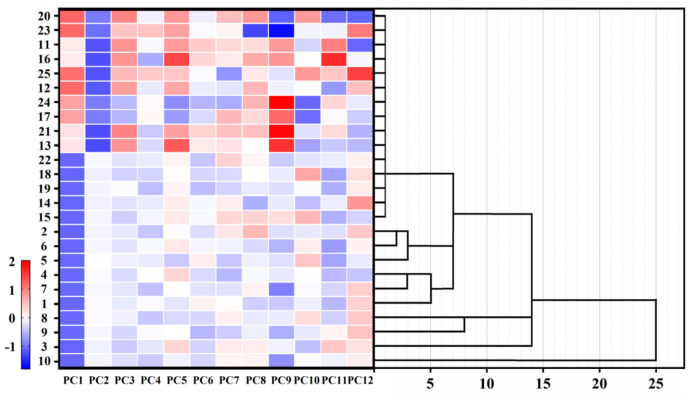
Hierarchical clustering performed with 25 abalone samples based on 5 standardized abalone.

To visualize the influence of MRP treatment on boiled, dried, and rehydrated abalone, complex relaxation results were transformed into a series of feature vectors. Dimensionality reduction was used to conveniently eliminate the excess T_2_ information [42], which was utilized to analyze the phase behavior and moisture stability in composite MP gelation [43]. Figure 7 shows the differences between treated and untreated abalones, where each row represents one sample and each column represents one clinical variable. The agglomerative hierarchical clustering algorithm was validated using indices including NMR and TPA data on 25 abalones and performed using 12 PC (principal component) variables. The first 12 PCs accounted for 90% of the variation in the relaxation delay curve for the first 2000 data, and correct classifications were achieved for 83% of the training set when 2 PCs were used. The red zones depicted in the heat map indicate relatively high PC values and are primarily located in the upper region of Figure 7; conversely, the blue zones suggest low PC values. The higher area with elevated values is associated with rehydrated abalone, while the lower area is predominantly linked to boiled samples. The heterogeneous T_2_ and TPA information was influenced by MRPs in abalone and subsequently normalized through dimensionality reduction techniques. Notably, the drying process exhibited significant specificity towards moisture levels in boiled and rehydrated abalone.

Artificial neural networks (ANNs) aim to uncover the intricate relationships between input and output parameters and to reveal the interconnections and network structures that exist between factors and outcomes. To differentiate dried abalones from non-dried ones treated or untreated with MRPs, PC1, PC2, PC3, and PC4 were utilized as input parameters to train an ANN model for predicting TPA parameters (Table 1). In this study, 20 experimental results were divided into a 7:3 ratio for training and validation purposes using a multilayer perceptron (MLP) developed by SPSS. Randomly assigned data groups were trained undifferentiated with the data processor to exclude the sensory parameters. The input parameters and output levels of the artificial neural network are shown in Table S1. The TPA results were categorized into high and low levels, and the relaxation decay was transformed into feature vectors for effective training.

**Figure 8:**
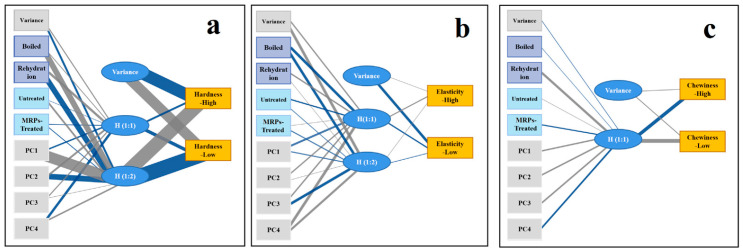
Correlations between different processes and (**a**) hardness, (**b**) elasticity, (**c**) chewiness of abalone.

Tables S2–S4 display the bonding values between TPA results and each factor calculation using the ANN. The predictive model was constructed with one input layer consisting of nine neurons and one accompanying variable, via a hidden layer including bonding values and one variance. The ANN work for the dependent variables was classified as high TPA or low TPA results and variance. In a more complex ANN model, the hidden layer of hardness and springiness consisted of three neurons. Figure 8a shows that boiling and rehydration processes had a stronger correlation with hardness. The bonding values between H(1:1)/H(1:2) and output neurons indicated that T_2_ relaxation was directly correlated with hardness, with values of 3.20 and −3.50. Similar results are demonstrated in Figure 8b,c, suggesting that rehydration exerted a more pronounced impact on chewiness and elasticity. In the hidden layer of the ANN model for chewiness and cohesiveness, two neurons were assumed for further analysis. Undoubtedly, moisture is a critical factor in TPA, particularly with regard to hardness and cohesiveness, and some degree of variance is inevitable.

## 4. Conclusions

In summary, MRPs emitting bright fluorescence were synthesized via a high-temperature and -pressure process using beef scraps as the base material. The prepared MRPs exhibited respectable fluorescence properties with chemical groups on their surface. Due to their small size and strong permeability, these MRPs are considered an effective additive for improving the moisture content and texture of processed abalone. The addition of MRPs had a greater effect on dried abalone, thus suggesting a promising approach to enhancing moisture distribution and texture parameters. This study demonstrated the practical application and quality enhancement of dried marine products through the innovative utilization of beef processing by-products.

## Figures and Tables

**Table 1:** TPA evaluation of abalone under different treatments.

	Characteristics	Con	Con-MRPs	MRPs
**Boiled**	Elasticity	0.85 ± 0.01 ab	-	0.89 ± 0.05 a
	Chewiness	4849.73 ± 274.63 b	-	5849.69 ± 961.05 a
	Hardness	8238.61 ± 391.13 ab	-	8568.76 ± 271.55 a
**Dried**	Elasticity	0.65 ± 0.02 a	-	0.63 ± 0.02 a
	Chewiness	6274.03 ± 637.60 a	-	5884.81 ± 1219.99 b
	Hardness	12,648.37 ± 513.86 b	-	14,460.47 ± 500.98 a
**Rehydration**	Elasticity	0.64 ± 0.03 b	0.65 ± 0.02 ab	0.67 ± 0.01 a
	Chewiness	6225.65 ± 529.51 ab	5979.24 ± 915.55 b	6582.27 ± 161.47 a
	Hardness	13,064.74 ± 932.46 a	13,209.61 ± 1866.91 a	10,640.57 ± 433.56 b

Different lowercase letters indicate significant differences between groups (*p* < 0.05).

## Data Availability

The original contributions presented in this study are included in the article/Supplementary Materials. Further inquiries can be directed to the corresponding author.

## Supplementary Materials

The following supporting information can be downloaded at https://www.mdpi.com/article/10.3390/foods14152726/s1, Figure S1: The cell viability of NRK cells subjected to different concentrations of MRPs for 12 h. Figure S2: Lipid oxidation in rehydrated abalone subjected to different treatments. Figure S3: T_2_ distribution of abalone treated or untreated with MRPs extracted from beef scrap. Table S1: ANN model for predicting TPA parameters in hierarchical clustering. Table S2: Input parameter and output levels for ANN work. Table S3: Bonding value of cohesiveness with each factor calculation using multilayer perceptron network. Table S4: Bonding value of chewiness with each factor calculation using multilayer perceptron network.
