# Supplementary material for: Mechanistic Insights into Nano-Maillard Reaction Products Regulating the Quality of Dried Abalones
Source: Foods. 2025 Aug 4;14(15):2726. doi: 10.3390/foods14152726 (PMC12346132; doi:10.3390/foods14152726)
Supplement: Supplementary file 1 [file foods-14-02726-s001.zip › foods-3778003-supplementary.pdf]

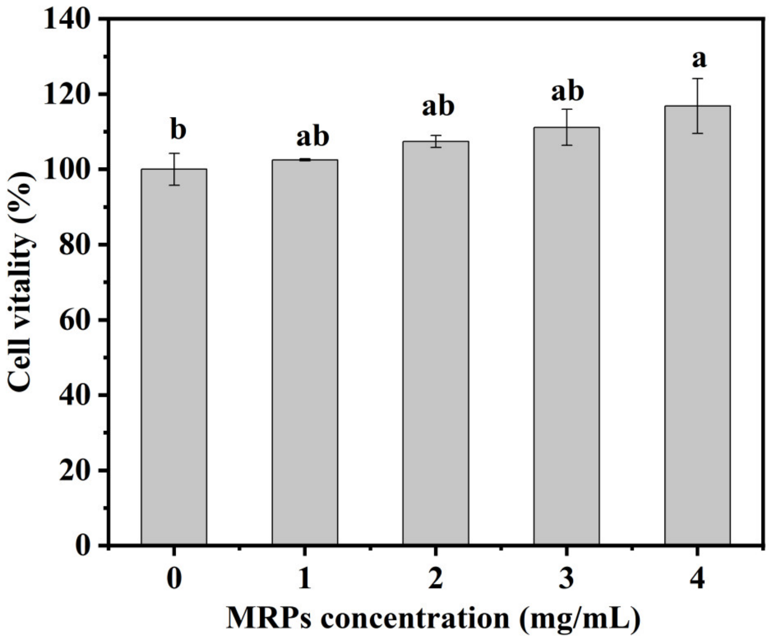

Figure S1. The cell viability of NRK cells after treatment with different concentrations of MRPs for 12 h

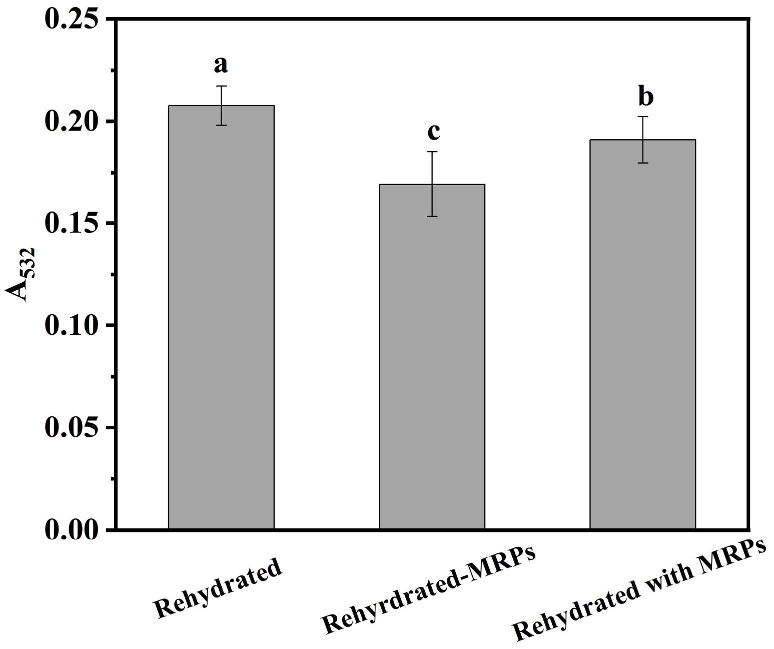

Figure S2. The evaluation of lipid oxidation in oysters subjected to different treatments

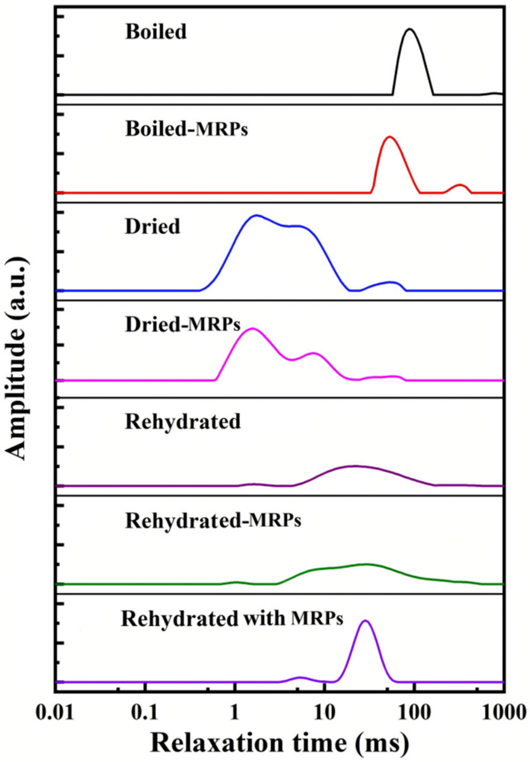

Figure S3. T_2 distribution processed abalone treated or untreated with MRPs extracted from beef scrap stock.

Table S1. ANN model for predicting TPA parameters in Hierarchical clustering**.**

	Factor	Level 1	Level 2
Input parameter	Boiled or rehdrated	Boiled	Rehdrated
	Untreated or MRPs treated	Untreated	MRPs treated
Output parameter	PC1-4	≥0	<0
	Hardness	≥6400	<6400
	Springiness	≥0.82	<0.82
	Chewiness	≥3500	<3500

Table S2. Bonding value of hardness with each factors calculation using multilayer perceptron network.

Hardness-Predicted value		Hidden layer		Output layer	
		H(1:1)	H(1:2)	High	Low
Input layer	Deviation	0.25	-0.46		
	Boiled	0.18	1.32		
	Rehydration	0.54	-1.28		
	Untreated	-0.01	0.41		
	Treated	-0.18	-0.18		
	PC1	-0.34	2.56		
	PC2	0.44	-1.33		
	PC3	0.29	0.11		
	PC4	-0.56	3.59		
Hidden layer	Deviation			-2.82	2.41
	H(1:1)			-0.45	-0.77
	H(1:2)			3.20	-3.50

Table S3. Bonding value of elasticity with each factors calculation using multilayer perceptron network.

Elasticity-Predicted value		Hidden layer		Output layer	
		H(1:1)	H(1:2)	High	Low
Input layer	Deviation	0.041	0.07		
	Boiled	-0.04	-0.13		
	Rehydration	0.36	0.36		
	Untreated	-0.34	-0.25		
	Treated	0.37	-0.35		
	PC1	-0.92	-0.36		
	PC2	0.54	0.11		
	PC3	0.42	-0.08		
	PC4	0.40	0.14		
Hidden layer	Deviation			0.02	-0.17
	H(1:1)			0.16	-0.46
	H(1:2)			0.36	-0.15

Table S4. Bonding value of chewiness with each factors calculation using multilayer perceptron network

Chewiness-Predicted value		Hidden layer	Output layer	
		H(1:1)	High	Low
Input layer	Deviation	-0.12		
	Boiled	-0.10		
	Rehydration	0.53		
	Untreated	0.03		
	Treated	-0.27		
	PC1	0.33		
	PC2	0.34		
	PC3	0.36		
	PC4	-1.04		
Hidden layer	Deviation		0.15	0.20
	H(1:1)		-0.74	0.88
